# *Ligustrum japonicum* Thunb. Fruits Exert Antiosteoporotic Properties in Bone Marrow-Derived Mesenchymal Stromal Cells via Regulation of Adipocyte and Osteoblast Differentiation

**DOI:** 10.1155/2021/8851884

**Published:** 2021-02-15

**Authors:** Jung Hwan Oh, Fatih Karadeniz, Jung Im Lee, Youngwan Seo, Chang-Suk Kong

**Affiliations:** ^1^Marine Biotechnology Center for Pharmaceuticals and Foods, College of Medical and Life Sciences, Silla University, Busan 46958, Republic of Korea; ^2^Division of Marine Bioscience, College of Ocean Science and Technology, Korea Maritime and Ocean University, Busan 49112, Republic of Korea; ^3^Department of Food and Nutrition, College of Medical and Life Sciences, Silla University, Busan 46958, Republic of Korea

## Abstract

*Ligustrum japonicum* fruits have been used as a part of traditional medicinal practices and supplements in Korea and Japan. It has been reported to possess various bioactivities, but its antiosteoporotic potential and active substances have not been reported yet. The present study followed an ALP activity and lipid accumulation-guided screening of *L. japonicum* fruits for antiosteoporotic compounds and isolated salidroside as an active compound. Antiosteoporotic effects of *L. japonicum* fruits and salidroside were examined in mesenchymal stromal cells by their ability to enhance osteoblast formation by increased ALP activity and osteogenic marker gene expression while suppressing adipogenesis by inhibition of lipid accumulation and adipocyte marker gene expressions. Results showed that salidroside was able to enhance osteoblast differentiation via Wnt/BMP signaling pathway overactivation and suppress the PPAR*γ*-mediated adipocyte differentiation, both through the MAPK pathway. In conclusion, *L. japonicum* fruits were suggested to possess antiosteoporotic activities and to be a source of antiosteoporotic substances such as salidroside.

## 1. Introduction

Bone-related complications and diseases cause low life quality, mortality, and morbidity for an increasing portion of the world population [1]. As the median age of most countries is rising steadily, age-related problems, including bone disorders, also appear in a similar increasing trend. Osteoporosis can be characterized with diminishing bone mass and as of today is one of the top concerns threatening the aging population. The bone fractures linked with osteoporosis are expected to be seen in 40% of women and 14% of men aged above 50 [2]. Currently, osteoporosis is treated mainly with drug cocktails and therapies, with bisphosphonates, vitamin D analogues, RANKL inhibitors, and estrogen therapy being the most common methods apart from weight-bearing exercises which is recommended as an important treatment for osteoporosis [3, 4]. The main idea behind the current osteoporosis treatment research is to ameliorate the deterioration of bone structure. Increasing bone tissue formation by tipping differentiation commitment of marrow mesenchymal cells towards osteoblasts rather than adipocytes is one of the ways to relieve the osteoporotic damages on bone. Increased differentiation and proliferation of osteoblasts help to relieve damaged bone mass balance [5]. However, complex interactions between osteoporosis progression, pharmacotherapy targets, and diabetes are a matter to be taken seriously for elder patients diagnosed with metabolic syndrome [6, 7]. Therefore, natural products and nutraceuticals are in the limelight for the development of drugs against osteoporosis given the exceptional chemical and structural diversity compared to synthetic libraries. Several studies showed that natural origin substances may have roles as enhancers of osteoblastogenesis *in vitro* [8, 9].

Furthermore, osteoporotic bone structure arises not only as decreased osteoblast formation but also increased adipogenesis [10]. Under osteoporotic conditions, bone marrow mesenchymal stromal cells increasingly commit to adipogenic lineage which is the reason behind the fracture-prone bone formation. Treatment for diabetic patients targets pathways that take role in differentiation mechanisms, especially adipogenesis which may be credited to be the cause of decrease in osteoblast differentiation [6]. Also, obesity induces adipokine formation which results in activation of osteoclasts in bone marrow hindering osteoblast formation [7]. Bone marrow-originated osteoblasts and adipocytes are differentiated from the same progenitor cells with antagonistic pathways. The regulation of the proliferator-activated receptor (PPAR) *γ* pathway is one of the common signaling cascades that govern adipogenic and osteoblast differentiation [11]. Activation of this pathway induces adipogenesis in bone marrow while suppression tilts the differentiation balance towards osteogenesis. On this ground, regulation of adipogenic and osteogenic differentiation homeostasis in bone is a promising therapeutic target against osteoporotic complications. Taken together, enhancing the osteogenic differentiation in bone marrow stromal cells accompanied with hindered adipogenic differentiation is a high-potential therapeutic approach for the treatment and prevention of osteoporosis in elderly population.

Plant metabolites allocate the reasonably high portion of natural therapeutic substances, especially against metabolic syndromes such as obesity, diabetes, cardiovascular diseases, and osteoporosis. Some profoundly studied phytochemicals with promising health beneficial effects are polyphenols, flavonoids, coumarins, caffeic acids, and their derivatives [12–14]. *Ligustrum japonicum* Thunb. (Waxleaf privet), inhabiting the southern coastal parts of Korea and Japan, is a subtropical evergreen small tree belonging to the *Oleaceae* family. It produces a small black fruit which has been found in traditional medicine practices as an ingredient against liver and kidney complications [15]. Major chemical components of the plants in the same genus include mannitol, glucose, palmitic acid, stearic acid, oleic acid, linolenic acid, and phenolic compounds such as catechin, quercetin, acteoside, aucubin, betulinic acid, oleanolic acid, and ursolic acid [16–18]. Fruits of *L. lucidum* were shown to yield salidroside [19], major constituent of *Rhodiola rosea* with various health benefits including osteoprotection [19, 20]. Salidroside was reported to possess osteoblast differentiation stimulation effect in mice [20]. In this context, the current study reported for the first time the effects of *L. japonicum* fruits on adipogenic and osteogenic differentiation of human bone marrow-derived mesenchymal stromal cells (hBM-MSCs). In addition, salidroside was isolated as one of the active constituents of *L. japonicum* fruits and its mechanism of action was studied in hBM-MSCs.

## 2. Materials and Methods

### 2.1. Plant Material and Isolation

**Figure 1:**
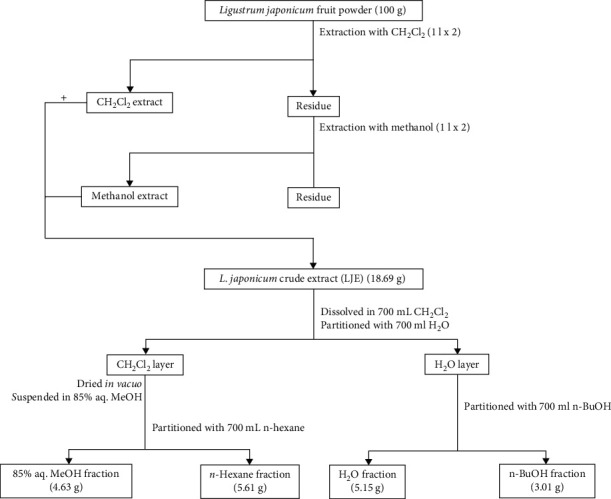
Extraction and solvent fractionation of *Ligustrum japonicum* Thunb. fruits.

The fruits of *L. japonicum* (500 g) were collected from Jeju Island (Rep. of Korea) in the fall of 2018 and separated from their stems and leaves. Fruits were sun-dried and ground to powder using a blender. The *L. japonicum* fruit powder (100 g) was extracted with 3 l of 100% dichloromethane (CH_2_Cl_2_), repeated three times. Remaining residue was then extracted with 3 l of methanol (100%) which was repeated three times as well. Methanolic and dichloromethane extracts were then combined to yield crude extract of *L. japonicum* fruits (LJE) (21.69 g). A stock solution of LJE (1 mg/ml) was prepared with 3 g of crude extract in 10% DMSO and used for further experiments. The remaining LJE (18.69 g) dissolved in distilled water (700 ml) was fractionated with the addition of CH_2_Cl_2_ (700 ml) using a separating funnel. CH_2_Cl_2_ layer was dried in vacuo, and obtained fraction was fractionated by dissolving in 85% aq. MeOH (700 ml) and addition of *n-*hexane (700 ml). On the other hand, the remaining H_2_O layer was separated by addition of *n-*BuOH (700 ml). Overall, the separation process yielded four fractions which were then dried in vacuo and named according to the solvent they were obtained from: *n-*hexane (5.61 g), 85% aq. MeOH (4.63 g), *n-*BuOH (3.01 g), and H_2_O (5.15 g). A scheme for the extraction and solvent fractionation is given in Figure 1. The compound used in the current study was isolated from the *n-*BuOH fraction following a column chromatography using stepwise gradient mixtures of H_2_O and MeOH (50%, 60%, 70%, 80%, and 90% aq. MeOH and 100% MeOH) run through R18 silica gel packing. Among subfractions, elute of 60% aq. MeOH was the source of the salidroside (10.4 mg) which was procured from 50% aq. MeOH elution. Qualitative evaluation of the salidroside was based on HPLC using YMC-Pack ODS-A column. All reagents for the extraction and isolation procedures were obtained from Sigma-Aldrich (St. Louis, MO, USA).

The spectroscopic characterization of the salidroside was carried out with ^1^H and ^13^C NMR analysis (Varian Mercury NMR 300; Varian, Palo Alto, CA, USA). NMR spectra were measured using standard Varian pulse sequence programs at 300 MHz and 75.5 MHz, respectively. All chemical shifts were recorded to the residual chloroform-d and methanol-d4 (Cambridge Isotope Laboratories, Inc., Cambridge, MA, USA) peaks.

### 2.2. Cell Culture and Differentiation

Bone marrow-derived human mesenchymal stromal cells from a healthy male donor aged 64 were procured from PromoCell (hBM-MSC, C-12974). Initially cryopreserved cells were thawed in a 37°C water bath according to the manufacturer's manual and seeded in two T-75 flasks (#156472, Thermo Fisher Scientific, Rockford, IL, USA) using Mesenchymal Stem Cell Growth Medium 2 (C-28009, PromoCell). Incubation of the plates was carried out in an environment with 37°C temperature and 5% CO_2_ atmosphere. Subcultivation was performed according to the manufacturer's manual using Accutase solution (C-41310, PromoCell) and phosphate buffer saline (PBS; no. 10010023, Thermo Fisher Scientific). For osteogenic differentiation of hBM-MSC, cells at the passage number 3 or 4 were seeded at 6-well plates (1 × 10^5^ cells/well) coated with bovine fibronectin (10 *μ*g/ml; C-43050, PromoCell) and grown to confluence. Following 100% confluence, the cell culture medium was swapped with Mesenchymal Stem Cell Osteogenic Differentiation Medium (C-28013, PromoCell). Next, cells were incubated for 14 days (unless otherwise noted), changing the medium every third day without disturbing cell monolayer. LJE, its solvent fractions, and salidroside were supplied along initial differentiation inducement only along with the differentiation medium and were not present in subsequent medium changes. Also, a blank group was cultured without differentiation inducement and fed with Mesenchymal Stem Cell Growth Medium 2 during incubation. A control group was induced to differentiate without sample treatment. In case of adipogenic differentiation of hBM-MSCs, the same procedure was followed except the cells were induced to differentiate at 90% confluence replacing differentiation-inducing medium with Mesenchymal Stem Cell Adipogenic Differentiation Medium 2 (C-28016, PromoCell).

### 2.3. Cell Viability Assay

The effect of LJE, its solvent fractions, and salidroside on the viability of hBM-MSCs was investigated using MTT assay. Cells were seeded in 96-well plates (1 × 10^3^ cell/well) and incubated for 24 h which was followed by the sample treatment. Viability of the sample-treated and untreated cells (in culture medium) was assessed after 72 h incubation. In the case of salidroside, the viability of vehicle only-treated cells was also analyzed compared to untreated control (in culture medium) to confirm the effects of treatment vehicle on cell viability. Briefly, cell wells were aspirated after treatment and wells were supplied with 100 *μ*l of MTT reagent (1 mg/ml; M2128, Sigma-Aldrich). Plates were then kept 4 more hours at 37°C incubator. Viable cell-dependent formation of formazan salts was quantified by addition of 100 *μ*l DMSO (100%) to each well and measurement of the absorbance value at 560 nm with a microplate reader (Multiskan GO, Tecan Austria GmbH, Grodig, Austria).

Proliferation of the osteo-induced hBM-MSCs was investigated using MTT assay. Cells were seeded in 6-well plates (1 × 10^5^ cells/well) and osteo-induced as stated earlier. Osteo-induced and noninduced cells were incubated 5 days with or without samples. After 5 days, viability of the cells was quantified using MTT assay as described above.

### 2.4. Staining of Intracellular Lipids with Oil Red O

Lipid accumulation by adipo-induced hBM-MSCs was observed using Oil Red O staining. Briefly, cells were cultured in 6-well plates (1 × 10^5^ cells/well) and differentiated into adipocytes as described earlier. Following 14 days of differentiation, wells were aspirated and washed with PBS followed by cell fixation via addition of 10% formaldehyde solution (in PBS, *v*/*v*; #252549, Sigma-Aldrich). Following 1 h of fixation, wells were washed with distilled water and the staining of the lipid droplets was performed by the addition of 0.5% 1 ml Oil Red O solution (*m*/*v*, 60% isopropanol and 40% water) into wells. After 1 h, Oil Red O staining solution was removed, wells were intensively washed with distilled water to remove the unbound dye, and plates were air-dried at room temperate for 1 more hour. Stained lipid droplets were visualized under an optical microscope (Olympus, Tokyo, Japan). Stain from the lipid droplets was eluted by adding 1 ml 100% isopropyl alcohol to wells. Quantification was carried out by measuring the absorbance values of elutes at 500 nm using a microplate reader (Multiskan GO).

### 2.5. Cellular Alkaline Phosphatase (ALP) Activity

ALP activity was evaluated in osteo-induced hBM-MSCs at day 7 of differentiation. Cells were seeded in 6-well plates (1 × 10^5^ cells/well) and osteo-induced as previously described. At day 7 of differentiation, wells were washed with PBS and the cells were lysed with the addition of 1 ml 0.1% Triton X-100 (T9284, Sigma-Aldrich) in 25 mM carbonate buffer. The lysates were then centrifuged for 15 min at 4°C (12,000 × *g*). The cellular ALP activity was assessed using the supernatants. Total protein content of the lysates was measured with a BCA protein assay kit (#23227, Thermo Fisher Scientific). Analysis of ALP activity was performed using a commercial kit (K412-500; BioVision, Hannover, Germany) following the producer's instructions.

### 2.6. Alizarin Red Staining

Extracellular calcium deposits of osteo-induced hBM-MSCs were observed by Alizarin Red staining. Cells were cultured in 6-well plates (1 × 10^5^ cells/well) and osteo-induced as previously described. At day 14 of differentiation, hBM-MSC osteoblasts were fixed on wells by addition of 1 ml ice-cold 70% EtOH at 4°C after aspirating the medium and washing wells with PBS. After 1 h, EtOH was removed from wells and cells were washed with distilled water. Alizarin Red S (A5533, Sigma-Aldrich) dyeing solution (pH 4.2, 2% *w*/*v* in distilled water) was then added to each well (1 ml/well), and the plates were kept at room temperature for 10 min in the dark. After staining, wells were aspirated, and cells were washed four times with 1 ml distilled water. Images of cells were taken by an Olympus microscope (Tokyo, Japan). Subsequently, the Alizarin Red dye was eluted from wells with 10% (*m*/*v*) cetylpyridinium chloride (C0732, Sigma-Aldrich) in 10 mM sodium phosphate buffer (P5244, Sigma-Aldrich) solution and mineralization was quantified by absorbance values at 560 nm using a Multiskan GO microplate reader (Tecan Austria GmbH).

### 2.7. Analysis of mRNA Expression

Cells were cultured in 6-well plates (1 × 10^5^ cells/well) and differentiated as previously described. Total RNA was isolated from differentiated and nondifferentiated hBM-MSCs at day 14 of differentiation using AccuPrep® Universal RNA Extraction Kit (Bioneer, Daejeon, Korea) according to the manufacturer's instructions. Total RNA was treated with RNase-free DNase I (EN0521, Thermo Fisher Scientific). The cDNA synthesis from total RNA was performed using CellScript All-in-One cDNA Synthesis Master Mix (CellSafe, Yongin, Korea) following the producer's directions. Amplification of reverse-transcribed cDNA was carried out using AccuPower® PCR PreMix (Bioneer) and gene specific forward and reverse primers as previously reported [21]. The PCR amplification was carried out with an initial denaturation at 95°C for 1 min, followed by 40 PCR cycles, each cycle consisting of 95°C for 45 s and 60°C for 30 s. Detection of genes was accomplished by agarose (1.5% *w*/*v*) gel electrophoresis (30 min at 100 V). Gels were stained by keeping in ethidium bromide solution (0.1% *w*/*v*) and imaged with CAS-400SM Davinch-Chemi Imager™ (Davinch-K, Seoul, Korea). The mRNA levels were calculated by the densitometric measurement of the bands using ImageJ software [22]. *β*-Actin was used as an internal loading control.

### 2.8. Western Blotting

Detection of protein levels was performed using standard Western blotting. Briefly, cells were cultured in 6-well plates (1 × 10^5^ cells/well) and differentiated as previously described. Total protein was then extracted from cells treated with or without samples by lysing using 1 ml RIPA buffer (R0278, Sigma-Aldrich) and vigorous pipetting. Protein content of the lysates was measured with a BCA protein assay kit (#23227, Thermo Fisher Scientific). A part of total protein lysate (20 *μ*g) was separated by SDS-PAGE (4% stacking and 10% separating gels). Proteins on gels were transferred to polyvinylidene fluoride membrane (#15219904, Cytiva, Westborough, MA, USA) for immunoblotting. Blocking of membranes was carried out by keeping membranes in 5% skim milk (*v*/*v*) prepared in TBST buffer (20 mM Tris, 150 mM NaCl, 0.1% Tween 20 at pH 7.5) for 4 h on a shaking incubator. Blocked membrane was hybridized at 4°C overnight with primary antibodies (1 : 1000 in 1x TBST buffer containing 5% bovine serum albumin (*m*/*v*)) against PPAR*γ* (#2443; Cell Signaling Technology, Danvers, MA, USA), CCAAT/enhancer-binding protein (C/EBP) *α* (#2295; Cell Signaling Technology), sterol regulatory element-binding protein-1c (SREBP-1c) (ab3259; Abcam, Cambridge, England, UK), ALP (ab65834; Abcam), BMP-2 (ab14933; Abcam), osteocalcin (ab93876; Abcam), RUNX2 (ab23981; Abcam), Smad1 (sc-7965; Santa Cruz Biotechnology, Santa Cruz, CA, USA), phospho(p-)Smad1/5 (#9516; Cell Signaling Technology), Wnt 10b (sc-25524; Santa Cruz Biotechnology), *β*-catenin (#9562; Cell Signaling Technology), p-*β*-catenin (#4179; Cell Signaling Technology), Axin (sc-14029; Santa Cruz Biotechnology), p38 (#8690; Cell Signaling Technology), p-p38 (#4511; Cell Signaling Technology), JNK (LF-PA0047; Thermo Fisher Scientific), p-JNK (sc-293136; Santa Cruz Biotechnology), ERK (#4695; Cell Signaling Technology), p-ERK (#4370; Cell Signaling Technology), AMPK (#2603; Cell Signaling Technology), p-AMPK (#2531; Cell Signaling Technology), and *β*-actin (sc-47778; Santa Cruz Biotechnology). Next, membrane was incubated for 2 h at room temperature with horseradish peroxidase-conjugated secondary antibodies specific to primary antibodies (anti-mouse: #7076, anti-rabbit: #7074; Cell Signaling Technology). Membranes were stained with Amersham ECL Western Blotting detection kit (RPN2108, Cytiva) according to the manufacturer's instructions, and the protein bands were imaged with CAS-400SM Davinch-Chemi Imager™ (Davinch-K). Quantification was carried out by densitometric analysis of the bands using ImageJ software [22].

### 2.9. Statistical Analysis

Numerical results were given as average of three independent experiments ± SD run in triplicates where applicable unless otherwise noted. Groups in the same data series were subjected to one-way analysis of variance (ANOVA) with post hoc Duncan's multiple range test for statistical analysis (SAS v9.1, SAS Institute, Cary, NC, USA), and differences were defined significant at *p* < 0.05 level.

## 3. Results

### 3.1. Effect of *L. japonicum* Fruit Extract on Adipogenic and Osteoblastogenic Differentiation of hBM-MSCs

**Figure 2:**
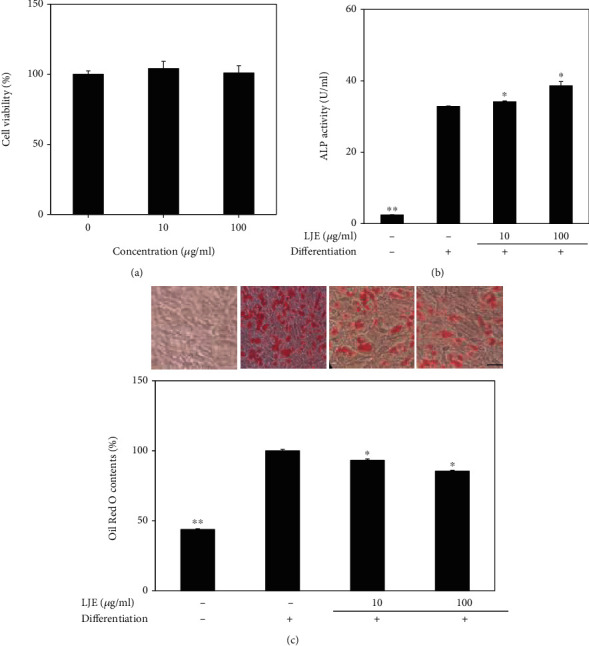
Effect of *L. japonicum* Thunb. fruit crude extract (LJE) on the viability of hBM-MSCs (a). Cells were seeded (1 × 10^3^ cells/well) in 96-well plates and incubated for 72 h untreated or treated with different concentrations (10 and 100 *μ*g/ml) of LJE. Viability of the cells was measured by quantification of MTT dye removed from cells. Cell viability is given as relative percentage of untreated control. Effect of LJE on the activity of cellular ALP (b). Cellular ALP activity of osteo-induced hBM-MSCs was measured with a spectrophotometric enzymatic activity assay at day 7 of differentiation. Effect of LJE on the intracellular lipid accumulation of adipo-induced hBM-MSCs (c). Following 14 days of differentiation, lipid droplets in adipo-induced hBM-MSCs were visualized with Oil Red O staining. Images of cells show stained lipid droplets (upper panel). Lipid accumulation levels were calculated by the colorimetric quantification of the dye removed from the wells (lower panel) and given as percentage of adipo-induced untreated control group. ^∗^*p* < 0.05 and ^∗∗^*p* < 0.01 vs. the untreated differentiated control group. Scale bar: 50 *μ*m.

The effect of *L. japonicum* fruit crude extract (LJE) on osteogenic and adipogenic differentiation was examined by ALP activity and lipid accumulation using human bone marrow-derived mesenchymal stromal cells. Increase in ALP production and accumulation of intracellular triglycerides are well-known markers of osteoblastogenesis and adipogenesis, respectively. Prior to evaluating the effects of LJE on differentiation of hBM-MSCs, its cytotoxicity was analyzed by MTT assay. As shown in Figure 2(a), 10 and 100 *μ*g/ml LJE treatment did not decrease the viability of hBM-MSCs, rendering the concentrations up to 100 *μ*g/ml safe to use for further assays. ALP activity was significantly stimulated in osteo-induced hBM-MSCs as expected and the presence of 100 *μ*g/ml LJE increased ALP activity to 38.62 U/ml from 33.99 U/ml of the untreated control group (Figure 2(b)). This increase in ALP activity suggested the possibility of osteoblastogenesis-enhancing substances in the fruits of *L. japonicum*.

The effect of LJE on the adipogenic differentiation of hBM-MSCs was screened by the intracellular lipid accumulation. As seen in Figure 2(c), during adipogenesis, intracellular lipid amount was increased by 128% compared to nondifferentiated cells. JLE inhibited the lipid accumulation in a concentration-dependent manner in differentiated adipocytes by 6.81 and 14.53% of the untreated control group at 10 and 100 *μ*g/ml concentrations, respectively, suggesting the presence of inhibitors for adipogenesis in LJE.

**Figure 3:**
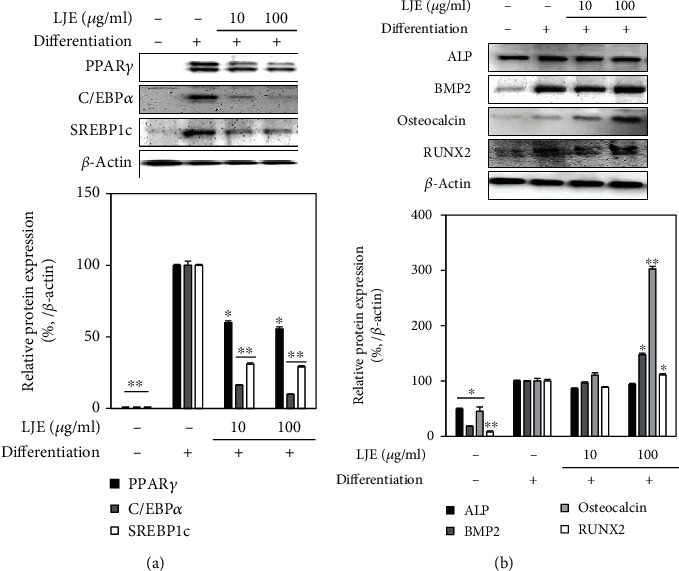
Effect of LJE on protein expression of (a) adipogenic and (b) osteogenic markers and transcription factors. Analysis of protein levels was carried out by Western blotting using the differentiated hBM-MSC lysates with or without LJE treatment (10 and 100 *μ*g/ml). Western blot bands were densitometrically quantified and plotted as relative percentage of the untreated differentiated control group. *β*-Actin was used as internal loading control and the expression. ^∗^*p* < 0.05 and ^∗∗^*p* < 0.01 vs. the untreated differentiated control group.

In order to evaluate the effects of LJE on the osteogenic and adipogenic pathways of hBM-MSC differentiation, key markers were analyzed at the protein level.  Figure 3(a) shows that LJE upregulated the protein expression of osteoblast differentiation markers BMP2 and osteocalcin significantly at 100 *μ*g/ml concentration. Additionally, at the same concentration, LJE was able to increase the protein levels of transcription factor Runx2 which is the key osteogenic marker for stromal cell differentiation. However, LJE did not significantly affect the protein level of ALP, although previous results indicated that it stimulated the ALP activity in osteo-induced hBM-MSCs. LJE treatment suppressed the protein levels of PPAR*γ*, C/EBP*α*, and SREBP1c, adipogenesis markers, and downstream activators for PPAR*γ*-regulated differentiation, in a dose-dependent manner (Figure 3(b)).

Results indicated that LJE potentially contained compounds with osteoblastogenesis-enhancing properties while suppressing the adipogenic differentiation of mesenchymal stromal cells. In this manner, a bioactivity-guided assay was carried out to investigate and elucidate the active ingredients. LJE was subjected to organic solvent fractionation and divided into four fractions, namely, H_2_O, *n-*BuOH, 85% aq. MeOH, and *n-*hexane fractions named according to the organic solvents from the fractions obtained.

### 3.2. Effect of Solvent Fractions of *L. japonicum* Extract on Adipogenic and Osteoblastogenic Differentiation of hBM-MSCs

**Figure 4:**
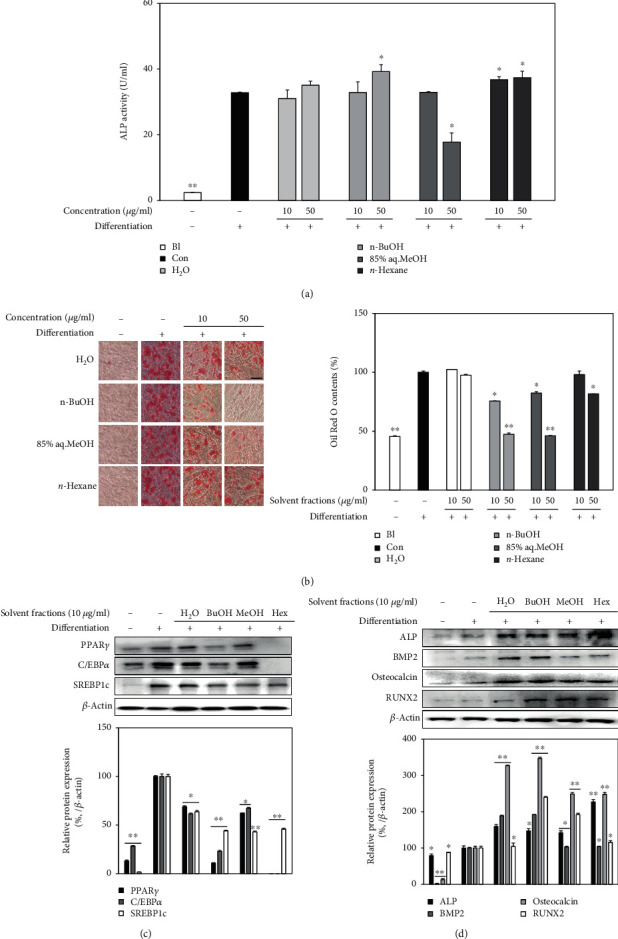
Effects of solvent fractions (H_2_O, 85% aq. MeOH, *n*-BuOH, and *n*-hexane) on adipogenic and osteogenic differentiation of hBM-MSCs. (a) hBM-MSCs were seeded in 6-well plates and osteo-induced with or without fractions (10 and 50 *μ*g/ml). Cellular ALP activity of osteo-induced hBM-MSCs was measured with a spectrophotometric enzymatic activity assay at day 7 of differentiation. (b) Adipo-induced hBM-MSCs in 6-well plates with or without fractions (10 and 50 *μ*g/ml) were stained with Oil Red O at day 14 of differentiation. Images of cells show stained lipid droplets (left panel). Lipid accumulation levels were calculated by the colorimetric quantification of the dye removed from the wells (right panel) and given as percentage of the untreated differentiated control group. (c) Adipogenic and (d) osteogenic protein levels were analyzed with Western blotting. Western blot bands were densitometrically quantified and plotted as relative percentage of the untreated differentiated control group. *β*-Actin was used as internal loading control. ^∗^*p* < 0.05 and ^∗∗^*p* < 0.01 vs. the untreated differentiated control group. Scale bar: 50 *μ*m.

Each solvent fraction from LJE was subjected to the same screening assays with LJE in order to evaluate their ability to enhance osteogenic differentiation of hBM-MSCs and suppress the adipogenesis. As shown in Figure 4(a), *n-*BuOH fraction at the concentration of 50 *μ*g/ml increased the ALP activity to 39.26 U/ml compared to 32.84 U/ml of osteo-induced untreated control hBM-MSCs, being the most active fraction in enhancing ALP activity. Other fractions except 85% aq. MeOH slightly increased the ALP activity while 85% aq. MeOH fraction expressed a notable inhibitory action. A similar trend was observed in lipid accumulation analysis of adipo-induced hBM-MSCs treated with LJE fractions. Among all fractions, *n-*BuOH and 85% aq. MeOH lowered the intracellular lipid amount by 52.62 and 53.94% at 50 *μ*g/ml dose, respectively, compared to untreated adipocytes (Figure 4(b)). For comparison, H_2_O and *n-*hexane fraction decreased lipid accumulation by 2.44 and 18.30% at the same concentration. Analysis of protein expression of osteogenic and adipogenic markers yielded similar results where *n-*BuOH was the most active fraction. Treatment with 10 *μ*g/ml *n-*BuOH fraction significantly suppressed the expression of PPAR*γ*, C/EBP*α*, and SREBP1c levels while stimulating the osteogenic markers such as ALP, BMP2, osteocalcin, and RUNX2 (Figures 4(c) and 4(d)). Parallel to ALP activity results, 85% aq. MeOH fraction showed inhibitory effects on osteogenic and adipogenic markers.

**Figure 5:**
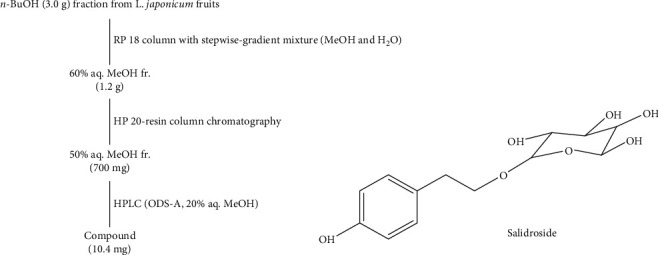
Isolation scheme and chemical structure of salidroside.

Overall evaluation of the results indicated *n-*BuOH fraction was the most active fraction to enhance osteoblast differentiation while suppressing the adipocyte differentiation of hBM-MSCs. Considering its notably higher activity, *n-*BuOH fraction was applied to further isolation processes to yield a known compound, salidroside, as one of the active constituents of the *L. japonicum* fruits. The chemical structure of the salidroside was elucidated by extensive 2D NMR experiments such as ^1^H COSY, NOESY, HSQC, and HMBC as well as by comparison with the published spectral data as described earlier (Figure 5) [23].

### 3.3. Effects of Salidroside on the Osteoblast Differentiation of hBM-MSCs

**Figure 6:**
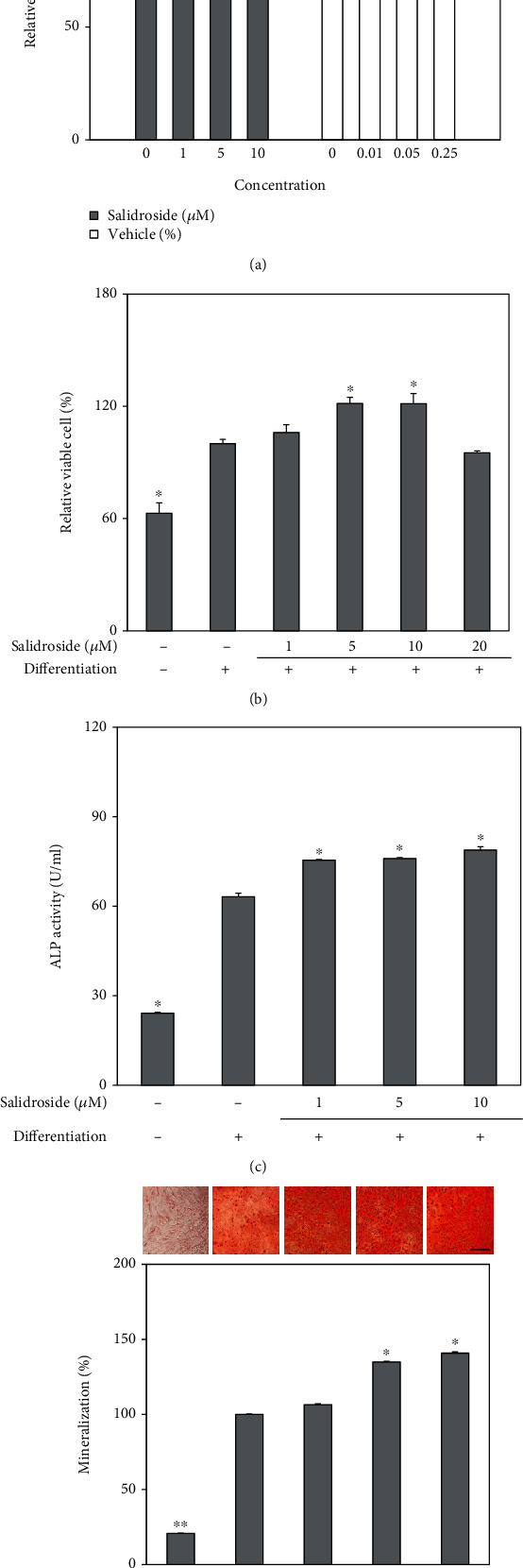
Effect of salidroside on the osteogenic differentiation of hBM-MSCs. Viability of the (a) noninduced and (b) osteo-induced cells was measured after 72 h and 5 days, respectively. Cells were seeded (1 × 10^3^) in 96-well plates with different concentrations (0, 1, 5, and 10 *μ*M) of salidroside and 10% DMSO as vehicle (0, 0.01, 0.05, and 0.25% final concentrations). Cell viability was compared to untreated control kept in culture medium only and given as relative percentage (a). hBM-MSCs were osteo-induced at 100% confluence without or with salidroside treatment (1, 5, and 10 *μ*M) (b). Effect of salidroside (1, 5, and 10 *μ*M) on the cellular ALP activity of osteo-induced hBM-MSCs was measured with a spectrophotometric enzymatic activity assay at day 7 of differentiation (c). Effect of salidroside on extracellular calcification was examined in osteo-induced hBM-MSCs without or with salidroside (1, 5, and 10 *μ*M) treatment (d). At day 14 of differentiation, hBM-MSCs were stained with Alizarin Red. Images of cells show stained calcium deposits (upper panel). Mineralization levels were calculated by the colorimetric quantification of the dye removed from the wells (lower panel) and given as percentage of the untreated differentiated control group. ^∗^*p* < 0.05 and ^∗∗^*p* < 0.01 vs. the untreated differentiated control group. Scale bar: 25 *μ*m.

First, the osteoblast differentiation stimulation properties of salidroside were examined with the same methods with LJE and its fractions. Salidroside was introduced to the osteo-induced and noninduced hBM-MSCs in different concentrations (1, 5, 10, and 20 *μ*M) and screened for its effect on the proliferation of the cells. Both salidroside treatment and equal amounts of vehicle (10% DMSO) without salidroside did not cause any cytotoxicity in the cells (Figure 6(a)). The cells were subjected to salidroside treatment for 48 h, and the viable cell amount was examined by MTT assay. Salidroside stimulated the viable cell amount which was suggested to be a result of an increase in proliferation of osteo-induced cells. Up to 10 *μ*M, the proliferation of the hBM-MSCs was increased in a slight dose-dependent manner where at 10 *μ*M treatment viable cell amount was 121% of untreated osteo-induced control (Figure 6(b)). However, at the concentration of 20 *μ*M, cell viability was dropped to a level 4.95% lower than that of the control group. This sharp decrease in cell viability at 20 *μ*M concentration was speculated to be observed due to various reasons including cytotoxicity at higher concentrations. Therefore, future experiments were conducted with doses at and below 10 *μ*M.

**Figure 7:**
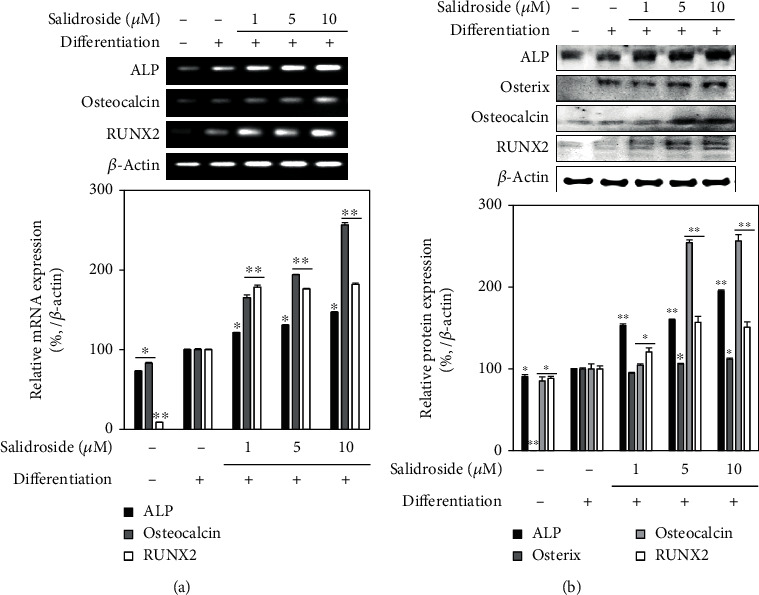
Effect of salidroside on (a) mRNA and (b) protein expression of osteogenic markers. Analysis of mRNA and protein levels was carried out by RT-PCR and Western blotting, respectively, using the osteo-induced hBM-MSC lysates. PCR and protein bands were densitometrically quantified and plotted as relative percentage of the untreated differentiated control group. *β*-Actin was used as internal loading control. ^∗^*p* < 0.05 and ^∗∗^*p* < 0.01 vs. the untreated differentiated control group.

Next, the effect of salidroside on the osteoblast differentiation of hBM-MSCs was examined by ALP activity and intracellular calcification. ALP activity is a studied observable marker of osteogenesis. As seen in Figure 6(c), ALP activity was increased to 63.17 U/ml on day 7 of osteo-inducement compared to 24.14 U/ml of noninduced hBM-MSCs. The presence of salidroside stimulated the ALP activity of osteo-induced cells, with 10 *μ*M salidroside increasing the activity to 78.87 U/ml. This effect of salidroside was accompanied by stimulated osteoblastic intracellular mineralization of hBM-MSCs observed at day 14 of osteo-inducement. Visualized by the images of hBM-MSCs stained by Alizarin Red, salidroside notably increased the stained calcification of the cells in a dose-dependent manner (Figure 6(d)). At 10 *μ*M concentration, salidroside increased the stained mineralization by 42.72% of the untreated osteo-induced control. Effects of salidroside on the osteoblast differentiation were also analyzed by measuring the mRNA and protein expression key osteoblast markers, ALP and osteocalcin, as well as osteogenic transcription factors Osterix and RUNX2. Both mRNA and protein expression levels of osteoblast markers and transcription factors were upregulated by salidroside in a dose-dependent manner (Figure 7). Results indicated that salidroside was able to stimulate osteoblast differentiation of hBM-MSCs.

### 3.4. Effects of Salidroside on Adipocyte Differentiation of hBM-MSCs

**Figure 8:**
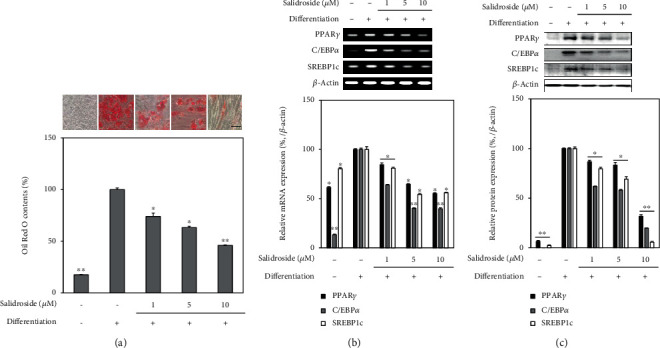
Effect of salidroside on the adipogenic differentiation of hBM-MSCs. (a) Adipo-induced hBM-MSCs in 6-well plates without or with salidroside treatment (1, 5, and 10 *μ*M) were stained with Oil Red O at day 14 of differentiation. Images of cells show stained lipid droplets (upper panel). Lipid accumulation levels were calculated by the colorimetric quantification of the dye removed from the wells (lower panel) and given as percentage of the untreated differentiated control group. Analysis of (b) mRNA and (c) protein levels was carried out by RT-PCR and Western blotting, respectively, using the adipo-induced hBM-MSC lysates at day 14 of differentiation. PCR and protein bands were densitometrically quantified and plotted as relative percentage of the untreated differentiated control group. *β*-Actin was used as internal loading control. ^∗^*p* < 0.05 and ^∗∗^*p* < 0.01 vs. the untreated differentiated control group. Scale bar: 50 *μ*m.

Screening of salidroside for adipogenesis inhibitory effect on adipo-induced hBM-MSCs was conducted via intracellular lipid accumulation evaluation. Cells were observed to accumulate lipid droplets as a marker for adipocyte differentiation. As seen in Figure 8(a), hBM-MSCs accumulated large amounts of lipid at day 14 of adipocyte differentiation. Lipid droplet levels, depicted as retained Oil Red O stain, were significantly reduced by salidroside treatment in a dose-dependent manner with 10 *μ*M salidroside treatment lowering the Oil Red O content by 53.96% of untreated adipocytes. Adipogenesis-inhibiting property of salidroside was further confirmed by the mRNA and protein levels of adipocyte markers and key adipogenesis transcription factors PPAR*γ*, SREBP1c, and C/EBP*α*. As shown in Figure 8(b), 10 *μ*M salidroside downregulated the expression of both mRNA and protein levels of adipogenesis-specific transcription factors. Overall, results showed that salidroside was one of the active ingredients of LJE.

### 3.5. Upregulation of Osteoblast Differentiation by Salidroside through the Wnt and BMP Signaling Pathway

**Figure 9:**
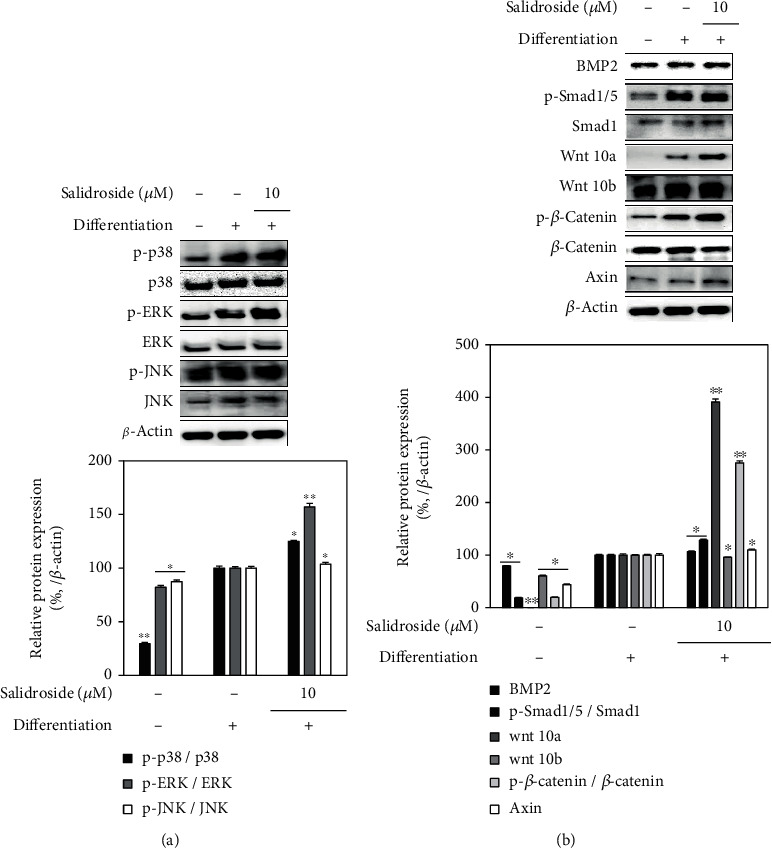
Effect of salidroside (10 *μ*M) on the levels of (a) p38, ERK, and JNK MAPKs and (b) Wnt/BMP signaling pathway. Analysis of protein levels was carried out by Western blotting using the osteo-induced hBM-MSC lysates at day 14 of differentiation with or without salidroside treatment. Western blot bands were densitometrically quantified and plotted as relative percentage of the untreated differentiated control group. Phosphorylation levels were measured via normalization of the phosphorylated (p-) protein against their total protein levels. *β*-Actin was used as internal loading control. ^∗^*p* < 0.05 and ^∗∗^*p* < 0.01 vs. the untreated differentiated control group.

Next, the effect of salidroside on the Wnt/BMP pathway was investigated. The effect of salidroside on the phosphorylation of mitogen-activated protein kinase (MAPK) proteins p38, ERK, and JNK was investigated. Results showed that at 10 *μ*M treatment, salidroside increased the phosphorylation of all three MAPKs (Figure 9(a)). These enhancing effect on the MAPK phosphorylation suggested that the salidroside enhanced osteogenesis via stimulation of MAPK-mediated activation of osteogenesis-specific gene expression. Considering the effect of salidroside on the expression of RUNX2 transcription factor, activation of *β*-catenin/Smad signaling was suggested to be enhanced by salidroside treatment. Salidroside (10 *μ*M) treatment enhanced the BMP and Wnt 10a levels (Figure 9(b)). Phosphorylated levels of BMP/Wnt downstream proteins Smad1/5 and *β*-catenin were also stimulated by the presence of salidroside (10 *μ*M). However, Wnt 10b levels were not affected by salidroside treatment. Increased levels of Axin by salidroside treatment also suggested that salidroside treatment enhanced the phosphorylation of Smad1/5 proteins. Overall, results indicated that salidroside stimulated BMP/Wnt signaling which in return stimulates osteogenic differentiation of hBM-MSCs.

### 3.6. Downregulation of Adipocyte Differentiation by Salidroside through 5′Adenosine Monophosphate-Activated Protein Kinase (AMPK)

**Figure 10:**
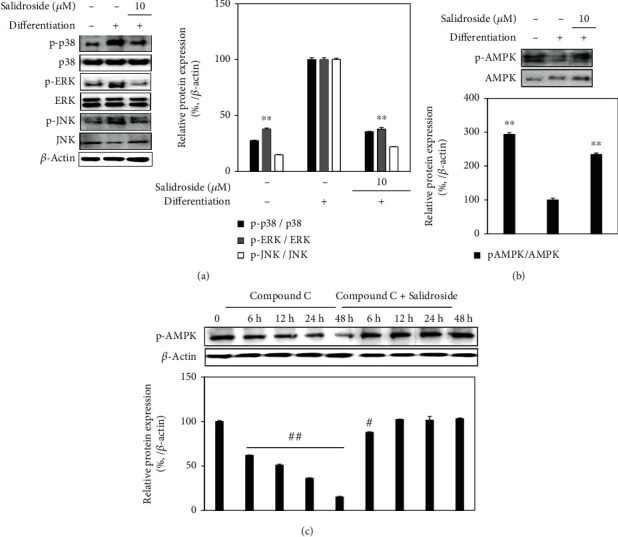
Effect of salidroside (10 *μ*M) on the levels of (a) p38, ERK, and JNK MAPKs and (b) AMPK. Phosphorylated (p-) and total protein levels analyzed by Western blotting using adipo-induced hBM-MSC lysates at day 14 of differentiation. (c) Time-dependent p-AMPK levels were obtained at the designated times from adipo-induced compound C-treated hBM-MSCs lysates with or without salidroside (10 *μ*M). Compound C was used as an inhibitor of AMPK phosphorylation. Western blot bands were densitometrically quantified and plotted as relative percentage of the untreated differentiated control group. Phosphorylation levels were measured via normalization of the phosphorylated (p-) protein against their total protein levels. *β*-Actin was used as internal loading control. ^∗^*p* < 0.05 and ^∗∗^*p* < 0.01 vs. the untreated differentiated control group; ^#^*p* < 0.05 and ^##^*p* < 0.01 vs. the differentiated control group without compound C and salidroside treatment.

Given that salidroside downregulated the PPAR*γ* and its downstream signaling proteins C/EBP*α* and SREBP1c in the assays stated previously, salidroside exerted this inhibitory effect via MAPK signaling cascade as it transacted in osteoblast differentiation. The inactive protein levels of p38, ERK, and JNK were not affected by salidroside treatment (10 *μ*M); however, phosphorylation of all three MAPKs was significantly inhibited (Figure 10(a)). Also, any involvement of salidroside in AMPK signaling was examined in a similar fashion. In Figure 10(b), treatment with 10 *μ*M upregulated both inactive and phosphorylated AMPK levels. This effect of salidroside on AMPK phosphorylation was further confirmed by using a specific AMPK inhibitor compound C. Introduction of compound C to adipo-induced hBM-MSCs time-dependently reduced the AMPK phosphorylation at 48 h. However, addition of salidroside relieved the inhibitory presence on AMPK phosphorylation as phosphorylated AMPK levels were significantly stimulated in a time-dependent manner despite compound C treatment (Figure 10(c)). Consequently, results suggested that salidroside inhibited adipogenesis in hBM-MSCs by suppressing MAPK activation and stimulating AMPK activation instead. Results altogether indicated that salidroside not only stimulates osteoblast differentiation but also prevents mesenchymal stromal cells to undergo excessive adipocyte differentiation. These properties of salidroside on hBM-MSC differentiation were suggested to stem from its ability to stimulate activation of BMP and Wnt signaling through MAPKs while suppressing PPAR*γ* signaling via upregulated AMPK activation.

## 4. Discussion

Bone marrow mesenchymal stromal cells are responsible for the bone tissue formation by committing to different cell lineages depending on the extracellular inducers. They are common progenitor cells for osteoblasts and adipocytes among others [24]. In osteoporotic conditions, it was shown that the balance between bone marrow adipocytes and osteoblasts could be changed in favor of adipocytes which results in diminished bone formation, softer bones, and linked complications [10]. In this context, aside from synthetic drugs, natural products have been studied as alternative sources for prevention and treatment of osteoporotic complications via enhancing osteoblast formation and inhibiting adipocyte differentiation [25, 26].

*L. japonicum* is a plant with various reported health benefits. In the present study, its effects on differentiation of hBM-MSCs were examined *in vitro*. The hBM-MSCs have the ability to differentiate into adipocytes and osteoblasts. Each differentiation progression starts with hBM-MSCs turning into preadipocytes or preosteoblasts, respectively. In the current study, hBM-MSCs were treated with LJE, fractions, and salidroside during the initial differentiation into preosteoblast and preadipocytes to analyze their effect on the regulation of differential tendencies. Successful differentiation into preosteoblasts is followed by osteoblast proliferation as a first step of bone formation and it is reported to be followed by increasing ALP activity for the extracellular matrix mineralization [27]. The preadipocyte MSCs differentiating into adipocytes are characterized by the expression of adipogenic genes which shows itself with intracellular lipid accumulation [28]. By using an ALP activity and lipid accumulation-guided extraction and isolation process, salidroside was obtained from crude extracts of *L. japonicum* fruits. In order to confirm the antiosteoporotic potential of salidroside, its effects were tested *in vitro* in adipo- and osteo-induced hBM-MSCs. Salidroside is one of the widely studied and recognized bioactive substances in *Rhodiola* plants and known for its antidepressant properties along with nervous system stimulation [29]. It was also isolated from different sources including *L. lucidum* [30]. The current study reported for the first time isolation of salidroside from *L. japonicum* fruits. Salidroside from *L. lucidum* had been reported to possess enhancing properties on osteoblast differentiation in murine MSCs via the BMP signaling pathway [31]. Also, antioxidant potential of salidroside was suggested to be one of the ways it might exert antiosteoporotic effect by reducing ROS in bone cells [32].

The present results showed that salidroside treatment enhanced the osteoblast differentiation in osteo-induced hBM-MSCs. Similar results were reported by Zhang et al. [32] in MC3T3-E1 mouse preosteoblasts where salidroside protected differentiating osteoblasts from oxidative stress-induced deterioration. However, the current study showed that salidroside could enhance osteoblast differentiation independent of elevated oxidative stress.

The differentiation of hBM-MSCs is regulated by the activation of pathways via MAPK-mediated phosphorylation [33]. In both osteogenesis and adipogenesis, MAPK signaling is activated which further phosphorylates and facilitates the nuclear translocation and transcriptional activity of transcription factors. PPAR*γ* and RUNX2 are two important transcription factors that regulate the differentiation of hBM-MSCs into adipocytes and osteoblasts, respectively. Results indicated that salidroside treatment suppressed the PPAR*γ* expression in adipo-induced hBM-MSCs while upregulating RUNX2 expression. Both transcription factors are phosphorylated by MAPKs prior to induce differentiation. Salidroside treatment was shown to have different effects on the phosphorylation of p38, ERK, and JNK MAPKs in adipocytes and osteoblasts. These MAPKs were shown to be inhibited by salidroside during adipogenesis. However, during osteoblastogenesis, salidroside treatment exerted an opposite effect, further increasing MAPK phosphorylation. Results showed that effects of salidroside on differentiation of hBM-MSCs did not affect the MAPK pathway specifically. Hence, it was suggested that salidroside played a role with the upstream effectors of MAPKs during adipogenic and osteogenic differentiation.

Osteoblast differentiation is evidently carried out by two main extracellular stimuli, BMP and Wnt [28]. The BMP pathway includes the downstream activation of Smad1/5/8 protein complex which subsequently facilitate the RUNX2-mediated expression of osteogenic genes. The BMP receptor-propagated signals also induce the phosphorylation of MAPK and osteoblast differentiation via non-Smad pathways [31]. Salidroside stimulated the phosphorylation of BMP-2 downstream target Smad1/5 protein along with MAPK activation. The Wnt/*β*-catenin signaling cascade was also shown to follow a similar path. Extracellular activation of Wnt receptors induces the phosphorylation of *β*-catenin and subsequent expression of osteogenic genes [33]. Among different Wnt subunits, Wnt 10a was shown to induce the activation of *β*-catenin via MAPK. As a downstream target for the Wnt pathway, *β*-catenin phosphorylation is crucial for Wnt-mediated osteoblast differentiation. Salidroside increased the phosphorylation of *β*-catenin and the levels of Wnt 10a. Taken together with its effect on BMP-2 levels, it was suggested that salidroside stimulated the osteoblast differentiation via its enhancing effect on BMP- and Wnt receptor-mediated signaling which in return increased both Smad and non-Smad (MAPK) phosphorylation.

Salidroside was already reported to inhibit adipogenesis in human visceral adipocytes through downregulation of the PPAR*γ* pathway [34]. The current results further confirmed that salidroside had similar effects in mesenchymal stromal cells. Similar to osteoblast differentiation, increased MAPK phosphorylation is observed during adipogenic differentiation as a result of PPAR*γ* ligand-mediated expression of adipogenic genes [35]. Salidroside suppressed the adipogenesis-induced activation of p38, ERK, and JNK phosphorylation which contradicts with the enhancing effect of salidroside on the MAPK activation during osteoblastogenesis. Vingtdeux et al. [36] reported that AMPK acts as a negative regulator for adipocyte differentiation. Activation of AMPK inhibits adipogenic gene expression and suppresses the phosphorylation of MAPKs during adipogenesis. Results showed that phosphorylation of AMPK was suppressed in untreated adipo-induced hBM-MSCs while MAPKs were actively phosphorylated. However, salidroside treatment relieved the suppression of AMPK activation. This was further confirmed with the addition of compound C, a small molecule AMPK inhibitor [37] along with differentiation of hBM-MSCs. Compound C-only treatment time-dependently suppressed the AMPK phosphorylation. Salidroside treatment reversed the effect of compound C on the AMPK activation. Results indicated that salidroside intervened with adipocyte differentiation of hBM-MSCs by stimulating the AMPK phosphorylation which explained its incompatible effect on MAPK activation.

On the other hand, the current study only investigated the effect of salidroside on the initial differential tendencies of hBM-MSCs given that samples were present during the early differentiation process and the cells were not treated during maturation. Future studies with longer treatment periods will provide insights towards the effects of LJE and salidroside on the osteoblast and adipocyte maturation. In this context, Guo et al. [38] reported that presence of salidroside stimulated the osteogenic differentiation of human MG-63 preosteoblast cells expressed as increased RUNX2 activity parallel to the present study. Furthermore, they also showed that salidroside treatment was able to facilitate fracture healing through enhancing HIF-1*α* expression modulated via the MAPK/ERK and PI3K/Akt signaling pathways. This was also confirmed by Li et al. [20] and Chen et al. [31] along with salidroside-stimulated activation of BMP signaling. The current results coupled with Guo et al. [38] study provide evidence that salidroside acts on MAPK signaling to enhance osteoblastogenesis. However, the stem cell line used in the current study was obtained from a single donor. Hence, it may lack the necessary data to overcome the donor-to-donor variations which might cause different profiles in MSCs. Therefore, future studies are urged where hBM-MSCs from multiple donors are used to eliminate the donor-donor variations and provide more evidence for the effect of samples on MSC differentiation.

## 5. Conclusions

In conclusion, the current findings demonstrated that *L. japonicum* fruits are a potential source of antiosteoporotic substances such as salidroside. Results showed that salidroside exerted capability to enhance bone formation in vitro. Salidroside treatment of the hBM-MSCs enhanced the osteogenic differentiation. Additionally, adipo-inducement of hBM-MSCs was suppressed by salidroside treatment. Effect of salidroside on the differentiation of hBM-MSCs was shown to arise depending on the extracellular stimuli. Osteo-inducement of the cells was enhanced by salidroside via Wnt/BMP signaling while adipo-inducement was hindered via phosphorylation of AMPK. All results considered, *L. japonicum* fruits and salidroside were suggested to be further investigated in animal models to develop novel antiosteoporotic compounds and confirm their antiosteoporotic capabilities and mechanisms.

## Data Availability

All data used to support the findings of this study are available from the corresponding author upon reasonable request.
